# Early Adverse Caregiving Experiences and Preschoolers' Current Attachment Affect Brain Responses during Facial Familiarity Processing: An ERP Study

**DOI:** 10.3389/fpsyg.2017.02047

**Published:** 2017-12-05

**Authors:** Melanie T. Kungl, Ina Bovenschen, Gottfried Spangler

**Affiliations:** Institute of Psychology, Friedrich-Alexander-Universität Erlangen-Nürnberg, Erlangen, Germany

**Keywords:** ERP, attachment, foster-care, facial familiarity, mother-child relationship, N170, Nc

## Abstract

When being placed into more benign environments like foster care, children from adverse rearing backgrounds are capable of forming attachment relationships to new caregivers within the first year of placement, while certain problematic social behaviors appear to be more persistent. Assuming that early averse experiences shape neural circuits underlying social behavior, neurophysiological studies on individual differences in early social-information processing have great informative value. More precisely, ERP studies have repeatedly shown face processing to be sensitive to experience especially regarding the caregiving background. However, studies on effects of early adverse caregiving experiences are restricted to children with a history of institutionalization. Also, no study has investigated effects of attachment security as a marker of the quality of the caregiver-child relationship. Thus, the current study asks how adverse caregiving experiences and attachment security to (new) caregivers affect early- and mid-latency ERPs sensitive to facial familiarity processing. Therefore, pre-school aged foster children during their second year within the foster home were compared to an age matched control group. Attachment was assessed using the AQS and neurophysiological data was collected during a passive viewing task presenting (foster) mother and stranger faces. Foster children were comparable to the control group with regard to attachment security. On a neurophysiological level, however, the foster group showed dampened N170 amplitudes for both face types. In both foster and control children, dampened N170 amplitudes were also found for stranger as compared to (foster) mother faces, and, for insecurely attached children as compared to securely attached children. This neural pattern may be viewed as a result of poorer social interactions earlier in life. Still, there was no effect on P1 amplitudes. Indicating heightened attentional processing, Nc amplitude responses to stranger faces were found to be enhanced in foster as compared to control children. Also, insecurely attached children allocated more attentional resources for the neural processing of mother faces. The study further confirms that early brain development is highly sensitive to the quality of caregiving. The findings are also relevant from a developmental perspective as miswiring of neural circuits may possibly play a critical role in children's psycho-social adjustment.

## Introduction

In the last decades, a growing body of research has focused on the effects of early adverse experiences on neurobiological functioning to explain psycho-social adjustment and later health outcomes (e.g., Pechtel and Pizzagalli, 2011). Pathogenic care puts children at high risk for maladjustment in several developmental domains and increases vulnerability to psychopathological outcomes in later life (e.g., Cicchetti, 2002). When being placed into more benign environments, however, many children are capable of forming secure attachments to their foster parents (Dozier et al., 2001; Stovall-McClough and Dozier, 2004; Cole, 2005; Oosterman and Schuengel, 2008; Ponciano, 2010; Jacobsen et al., 2014, see Caharel et al., 2005 for meta-analytic data). More precisely, attachment security has been found to significantly increase during the first year of placement (Gabler et al., 2014; Lang et al., 2016). Still, foster children have been found to lack an age-appropriate reticence around strangers, also referred to as disinhibited social engagement (Zeanah et al., 2004; Oosterman and Schuengel, 2008; Pears et al., 2010; Van Den Dries et al., 2012; Jonkman et al., 2014; Lawler et al., 2014), which tends to persist despite improvements in attachment security (Chisholm, 1998; Smyke et al., 2002; O'Connor et al., 2003; Rutter et al., 2007; Zimmermann, 2015). Recent evidence suggests that atypical brain activity (i.e., cortical hypoactivation) may be associated with this behavioral pattern (Tarullo et al., 2011; Mesquita et al., 2015). This emphasizes the role of neurophysiological studies when studying effects of early adverse experiences on behavioral development.

The current study follows the assumption that pathogenic care leads to alterations in neural circuits related to psycho-social functioning as biological systems develop in an adaptive response to the social environment (Cicchetti, 2002). Thereby, it focusses on an important aspect of social-information processing (i.e., face processing) and asks how it is affected by early adverse caregiving experiences and current attachment security.

### Theoretical background: important aspects of face processing

Inevitably, the processing of faces is an important aspect of any social interaction and numerous studies in infants and children provide evidence that the development of face recognition is highly influenced by early social experiences (see Moulson et al., 2009). For example, several studies could show that infants' regular interpersonal experiences facilitate the processing of faces they are exposed to frequently (e.g., Quinn et al., 2002). Also, the quality of the emotional environment has been shown to affect neural circuits leading to a facial emotion bias in maltreated children (e.g., Pine et al., 2005), and infants from non-abusive households (de Haan et al., 2004; Taylor-Colls and Fearon, 2015). This experience-dependent process allows for individual shaping of the face recognition system with regard to a given social environment (see Greenough et al., 1987; Nelson, 2001).

#### Face-sensitive ERP components in infancy and childhood

Due to its excellent temporal solution, the ERP method is a very practical approach to illustrate how neurocognitive processes in response to faces unfold over time (de Haan et al., 2007). We selected three well-studied components that can reliably be identified in preschoolers, to examine individual differences at different stages in the time-course of facial information processing (see Todd et al., 2008) in children with and without a history of pathogenic care.

#### P1

The first face-sensitive ERP component in the temporal sequence of neural face processing, the P1, occurs at occipital electrode sites approximately 100 ms following stimulus onset. It has repeatedly been reported in studies on face processing in infants (de Haan and Nelson, 1999), children (Taylor et al., 2001, 2004; Carver et al., 2003; Todd et al., 2008; Moulson et al., 2009; Mesquita et al., 2015), as well as adults (e.g., Halit et al., 2000; Itier, 2004), and is particularly likely to be sensitive to faces by 4 years of age (Taylor et al., 2001, 2004; Itier, 2004). The P1 might possibly reflect an early stage of facial encoding as it is sensitive to low-level individual differences between faces and non-face stimuli (Rossion and Caharel, 2011). Still, it is suggested that the activation of face representations in the brain does only appear at a later stage reflected by a subsequent negative component, the N170 (Rossion and Jacques, 2012).

#### N170

The N170 is an early-latency occipito-temporal ERP component and usually defined as the first negative peak following the P1. There is consistent evidence that the N170 is sensitive to faces as compared to non-face stimuli in adults (for an overview of the N170 “face effect” see Rossion and Jacques, 2012), and, occurring at longer latencies, in children by about 4 years of age (Taylor et al., 1999; Todd et al., 2008; Kuefner et al., 2010).

The N170's sensitivity to facial familiarity still seems to be a topic of debate. However, evidence suggests that personal importance of faces alters the N170 response by the activation of robust representations in the brain that have evolved due to frequent exposure (Caharel et al., 2002, 2005, 2006; Mesquita et al., 2015, but see Eimer, 2000). As an example, the N170 amplitudes was found to discriminate between mother's and random famous faces in adults (Caharel et al., 2005) and between caregiver and stranger faces in children (Todd et al., 2008; Moulson et al., 2009; Dai et al., 2014).

#### Nc

Finally, the Nc represents a well-studied ERP component in the face-processing literature. It is prominent in the fronto-central area of the scalp at mid-latencies. It is not specifically related to face-processing, rather does it appear to reflect the allocation of attentional resources in response to salient or interesting stimuli (Courchesne et al., 1981; Nelson, 1994; de Haan and Nelson, 1997; Reynolds and Richards, 2005). Thus, it is of major interest when studying the individual processing of faces that are assumed to vary in salience during the course of development. Indeed, the Nc component has, too, shown to discriminate between caregiver and stranger faces in infants and preschoolers (de Haan and Nelson, 1997, 1999; Dawson et al., 2002; Carver et al., 2003; Todd et al., 2008; Moulson et al., 2009; Webb et al., 2011).

### ERP evidence for individual differences in facial familiarity processing

#### Facial familiarity processing and the developing infant–caregiver relationship

The few ERP studies on facial familiarity processing in children give rise to the assumption that neural correlates of caregiver and stranger face processing clearly relate to important aspects of normative socio-emotional development. The Nc is of major interest as it is associated with attention to face stimulus. Interestingly, older children elicit larger Nc amplitude responses to a stranger's face as compared to the mother's face (Dawson et al., 2002; Carver et al., 2003; Todd et al., 2008; Moulson et al., 2009). Still, these neural correlates are suggested to vacillate frequently during the early years (Swingler, 2008). Interestingly, in infants, increased proximity seeking to the mother is associated with larger Nc amplitudes (heightened attentional processing) to stranger faces (Swingler et al., 2007). Thus, it is proposed that age-related changes in neural response patterns occur in conjunction with the formation of the child-caregiver attachment relationship (see Bowlby, 1982). And it also goes along with the child's attentional focus increasingly shifting from the mother to a broader social world (Carver et al., 2003; Swingler et al., 2007). Even though it has been proposed that social information processing varies as a function of attachment security (Dykas and Cassidy, 2011), however, to our best knowledge there is no study that has related the quality of the attachment relationship to mother and stranger face processing.

#### Facial familiarity processing in children from adverse rearing environments

Moulson et al. (2009) collected ERP data from currently institutionalized, never-institutionalized and previously institutionalized children placed in foster care while passively viewing pictures of their primary caregiver's and a stranger's face. First, they found institutionalized children to elicit smaller amplitudes than non-institutionalized children. Although the effect was only consistently present for the early visually evoked potential P1, waveforms indicated that later occipital components (P400, N170) were equally affected. This brain activity pattern is consistent with studies in children from low-SES backgrounds (Kishiyama et al., 2009) and non-ERP studies in children with a history of social deprivation (Otero et al., 2003; Marshall et al., 2004; Tarullo et al., 2011). In conclusion, it is claimed that a lack of an appropriately stimulating environment might lead to persistent cortical hypoarousal (also see Moulson et al., 2009). Second, previously institutionalized children placed in foster care did show some —but not full—recovery regarding the dampened ERP amplitudes to faces. However, there was no effect of timing of intervention on ERP outcomes (Moulson et al., 2009). Third, institutionalized and never-institutionalized children groups did hardly differ in response to the caregiver and stranger faces at later latencies despite their dramatically different caregiving backgrounds. Surprisingly, there were no significant interactions between group and facial type. However, findings were inconsistent across measurement points.

In a recent study, Mesquita et al. (2015) investigated children's facial familiarity processing in a sample of 3- to 6-year-old children currently living in Portuguese institutions. They addressed the non-significant interaction between group and facial type reported by Moulson et al. (2009) and extended the study by taking into account within group variations regarding disordered social behavior. Thereby, they found evidence that children displaying atypical social behavior are more likely than typically functioning children to elicit smaller P1 amplitudes in response to faces. Also, children with inhibited attachment symptoms appeared to elicit larger N170 amplitudes to their caregiver's face. Despite its exploratory nature, Mesquita et al.'s (2015) study suggests that it might not be institutionalization *per se*, but individual attachment related experiences and outcomes that alter children's neural processing of faces.

To conclude, all we know about the impact of adverse rearing environments on facial familiarity processing is based on these two studies. Thus, our knowledge exclusively stems from data assessed in (previously) institutionalized children. Also, associations found between children's behavior and brain activity solely rely on caregiver reports (i.e., Mesquita et al., 2015). Finally, there is a gap in the literature regarding studies that investigate overt attachment related behavioral and neural responses to familiar and unfamiliar persons in children with different family rearing backgrounds.

### The current study

Our first major aim was to compare foster children's neural processing of facial familiarity to an age matched control sample. Basic visual functions regarding face processing (as represented by the P1 amplitude response) were found to be impaired in institutionalized children (e.g., Moulson et al., 2009; Mesquita et al., 2015), but it is unclear whether the effect would show in children without a background of institutional rearing, since family and institutional rearing backgrounds clearly differ in terms of deprivation. Thus, we did not necessarily expect the P1 effect to be equally prominent in family reared foster children, still, we tested for the effect to confirm this assumption. In contrast, the N170 has shown to be affected by prolonged exposure to a particular face (e.g., Caharel et al., 2002). Thus, we expected the effect of facial familiarity, especially processing of the (foster) mothers face to vary with foster care status. Regarding the Nc, larger amplitudes relate to enhanced attentional processing of either the caregiver or the stranger's face depending on aspect of socio-emotional development (e.g., Carver et al., 2003; Swingler et al., 2007). Indeed, our previous findings suggest that during social interactions foster children seem to be more affected by a stranger's presence as observed on a behavioral level (Kungl, 2016). Thus, we expected Nc amplitude responses to stranger faces to be larger in foster children as compared to control children.

Our second major aim was to investigate effects of attachment security on facial familiarity processing in foster and control children. As suggested by Spangler and Zimmermann (1999) “including the physiological processes in addition to the psychological processes enables us” to gain further knowledge on “the function of the inner working model with respect to processes that are […] not expressed through overt behavior.” (p. 270). To our best knowledge, the studies conducted by Carver, Swingler, and their research group are the only studies relating changes in the attachment relationship (Carver et al., 2003) and attachment relevant behaviors (Swingler et al., 2007, 2010) to brain responses during mother-stranger face processing. Still, no study has looked at the quality of the attachment relationship as related to ERP data in children neither in high-risk nor in normative samples. Referring to the N170's sensitivity to facial familiarity, and thus, previous exposure to a particular face, we expected the quality of caregiving experiences (indexed by attachment security) to affect facial familiarity processing as early as the N170 time window. Securely attached children are by theory more likely to have frequently engaged in close social interactions, and thus face to face contact. This was expected to result in different N170 amplitude responses to faces. We further aimed to explore if there might be an interaction between foster care status and attachment security on N170 amplitudes. Also, as the attachment system is believed to regulate not only the child's psychological but also physiological processes related to his/her social world (e.g., Spangler and Grossmann, 1993; Spangler and Zimmermann, 1999), the attention drawn to familiar and unfamiliar faces, as represented by Nc amplitude responses, was expected to differ in securely attached children as compared to insecurely attached children.

## Methods

The current study is part of a larger project named “Attachment and Psychosocial Adjustment of Foster Children: Individual and Social Factors of Influence” (Spangler et al., 2009) that aimed to investigate preschool-aged foster children during the transition into the foster home. A detailed description of the longitudinal study as well as its findings can be found in other publications focusing on the development of attachment (Gabler et al., 2014; Lang et al., 2016) and attachment disorders (Zimmermann, 2015).

The foster group included in the current study forms a regional subsample of the overall study. Importantly, included children did not significantly differ from the rest of the sample regarding age at placement, attachment security, or problem behavior.

The current study reports on data retrieved at one home visit (for assessment of attachment security) and a laboratory visit^1^ (for ERP assessment) for both the control and the foster group. Prior to the assessments, informed consent entailing the purpose of the study, anticipated consequences, uses and storage of data as well as the voluntary basis of participation was negotiated with the (foster) mother and a written informed consent form was signed.

### Participants

The final foster sample included in the ERP analyses consisted of 13 foster children (8 male/5 female) and 24 children living in their birth family (10 male/14 female). Nine more children (incl. 3 foster children) were tested but excluded from further analyses due to technical problems (*n* = 3), non-compliance during the application of the electrode cap (*n* = 2), removal of the cap during the experiment (*n* = 1) or an insufficient number of artifact-free trials (*n* = 3).

At the ERP assessment foster children's age (months: *M* = 55.62, *SD* = 11.69) did not differ from control children's (months: *M* = 56.58, *SD* = 9.04), *t*_(35)_ = 0.28, *ns.*, and the time foster children had been living within the foster home was about 20 months (*M* = 20.23, *SD* = 5.00). In control children, attachment security was assessed within a few weeks after the ERP assessment (*M* = 1.59, *SD* = 0.84). In foster children, however, attachment security was assessed at an average of 8 months (*M* = 8.15, *SD* = 4.24) before the ERP assessment resembling the time point of 1 year (months: *M* = 12.62, *SD* = 0.91) after placement in the current foster home. Foster children's age at placement ranged from 15 to 61 months (*M* = 33.54, *SD* = 14.10).

### Measures

#### Attachment security

Children's attachment security was assessed during a 2.5 hrs home visit using the German version 3.2 of the Attachment Q-Sort (AQS; Waters and Deane, 1985, German version: Schölmerich and Leyendecker, 1999). The AQS is a widely used measure in studies with children aged 12–70 months (Ijzendoorn and Bakermans-Kranenburg, 2004). Conducting a series of meta-analyses Van Ijzendoorn et al. (2004) found evidence for the validity of the observer AQS as a measure of attachment. The original version of the AQS (Waters and Deane, 1985) was modified by Waters (1995) and includes 90 items referring to children's secure base and exploratory behavior as well as other aspects like social referencing. These items are individually printed on cards that are then sorted into 9 piles categorizing the child's behavior from “less characteristic” to most characteristic. Subsequently the child's Q-Sort is correlated with a hypothetical criterion sort developed by experts representing the prototypically behavior of a most secure subject (see Waters and Deane, 1985). In the present study, two trained observers separately sorted the Q-Set. Inter-observer reliability was high, *r* = 0.70 (range: *rs* = 0.39–0.87). Using a composite of both observers' Q-sets for the analyses enhanced reliability up to *r* = 0.82 as calculated by Spearman Brown formula. For the analysis, attachment security was dichotomized by median split.

#### Neurophysiological data

##### Procedure

The ERP assessment took place at the laboratory visit. Children were carefully instructed and introduced to the procedure in a child-oriented way. An electrode cap (ActiCap, Brain Products, Gilching, Germany)—referred to as being a “luminous magic cap”—was positioned after measuring the vertex and the experimenter injected gel in each electrode. Scalp impedances were indicated by color LEDs at the electrode. They were ideally kept below 15 kΩ, however 15–30 kΩ was graded acceptable in some cases. The average scalp impedance was 6.74 kΩ, (*SD* = 2.83 kΩ) across all participants.

The child was seated in front of a computer screen that was surrounded by a blue shielding to block the child's view of the room. While one experimenter was standing next to the child and led him/her through the session, another experimenter was sitting behind a curtain operating the recording and observing the child via a webcam. Eye movements and interruptions were marked online in the EEG. Children were told they should sit quietly and pay attention to their mother's^2^ as well as to a stranger's face, which would be shown on screen in a repeated fashion. A cartoon signaled the end of each block where children received tokens (Sticker) to maintain their motivation. These tokens were to be traded into a present afterwards.

##### Stimuli

Each child's mother was photographed at their visit to the laboratory. Pictures of their faces were taken against a blue background while sitting in an upright position and looking straight into the camera. Mothers were told to put on a friendly face but not to show teeth while smiling. Photographs were cropped and placed on a gray background layer. All color information was removed and images were adjusted in figure-ground ratio, position, contrast and lightning if necessary. In addition to the familiar face (mother) an unfamiliar face stimulus (stranger) was randomly selected from the pool of stimuli showing the other female participants. Pictures of mothers who wore glasses were always paired together. To maintain a foveal angle of 7.9° × 6.6°, and thus, minimizing eye movement picture size on screen was 9 × 7.5 cm at a distance of 65 cm.

##### EEG recording

EEG and EOG data were collected using 30 active electrode channels. Electrodes were placed in standard positions according to the international 10–20 system. EEG was recorded from Fp1, Fp2, Fz, F3, F4, F7, F8, FC6, Cz, C3, C4, T7, T8, CP1, CP2, CP5, CP6, TP9, TP10, Pz, P3, P4, P7, P8, Oz, O1, O2, PO9, PO10. One electrode was placed under the child's right eye and used for EOG recordings. Electrode AFz served as the ground and FCz as the online reference. The signal was continuously recorded with a sampling rate of 250 Hz (BrainAmp amplifier, Brain Products, Gilching, Germany). Stimuli were presented via a second computer using an experiment generating software (Inquisit 3, Millisecond Software, Seattle, Washington). Both computers were interfaced.

##### Assessment of event-related potentials

*ERP data collection*. ERPs were recorded while children were shown facial images of their mother and a stranger in a counterbalanced manner. Each trial consisted of a 300 ms fixation period during which a small yellow star appeared in the center of the screen followed by the presentation of the face stimulus for 700 ms and a post-stimulus recording period of 600 ms during which the screen was black. The subsequent inter-trial interval varied randomly between 500 and 1,000 ms. The presentation of stimuli was semi-randomized and the frequency of appearance was counterbalanced with 80 trials per face condition (mother, stranger). The experiment consisted of 8 blocks of 20 trials each allowing for small breaks in between blocks.

The average number of artifact-free trials was 38.97 (*SD* = 13.10) per condition. The number of trials did not significantly differ neither between conditions (*M*_*mother*_ = 37.97, *SD* = 13.93; *M*_*stranger*_ = 39.97, *SD* = 13.32), *t*_(36)_ = −1.62, *p* = ns, nor between groups (*M*_*controlgrou*__*p*_ = 41.31, *SD* = 13.79; *M*_*fostergrou*__*p*_ = 34.65, *SD* = 10.9), *t*_(35)_ = 1.50, *p* = ns. The number of trials is comparable to other studies using a similar paradigm in this age group (e.g., Carver et al., 2003).

*ERP editing and reduction*. Data was edited offline using BrainVision Analyzer (Version 2.4., Brain Products, Gilching, Germany). First, during raw data inspection intervals were eye movement artifacts occurred were taken out manually. Subsequently, a 0.3–30 Hz filter, and a 60 Hz notch filter (all having a 24 dB/oct gradient) were applied. When necessary bad channels were replaced via interpolation by spherical splines (order of splines = 4). The average number of replaced channels was 0.88 (*SD* = 1.66) across all participants. The continuous data was re-referenced to the average reference and the implicit online reference was reused as channel FCz. After segmenting data by face condition we ran an automatic artifact rejection not allowing for voltage steps of more than 100 μV, differences of two values in one segment of more than 150 μV and/or an amplitude exceeding 100 μV or falling below −100 μV, respectively. Since we showed a fixation cross for 300 ms prior to the actual stimulus we chose the interval from −500 to −300 ms for subsequent baseline correction. This way we ensured, that activity elicited by the fixation cross did not affect the actual baseline. Finally, a grand average for each face condition was calculated. Participants whose data provided less than 15 remaining segments per condition were not included in further analyses.

*ERP measures*. To detect components of interest we drew assumptions based on the literature and visually inspected the grand average waveforms and peak information (amplitude in mV) for each component and subject was extracted. Finally, mean amplitude values of ±1 sampling point around the designated individual peak were exported into the data analysing software SPSS 23.0.

**Figure 1:**
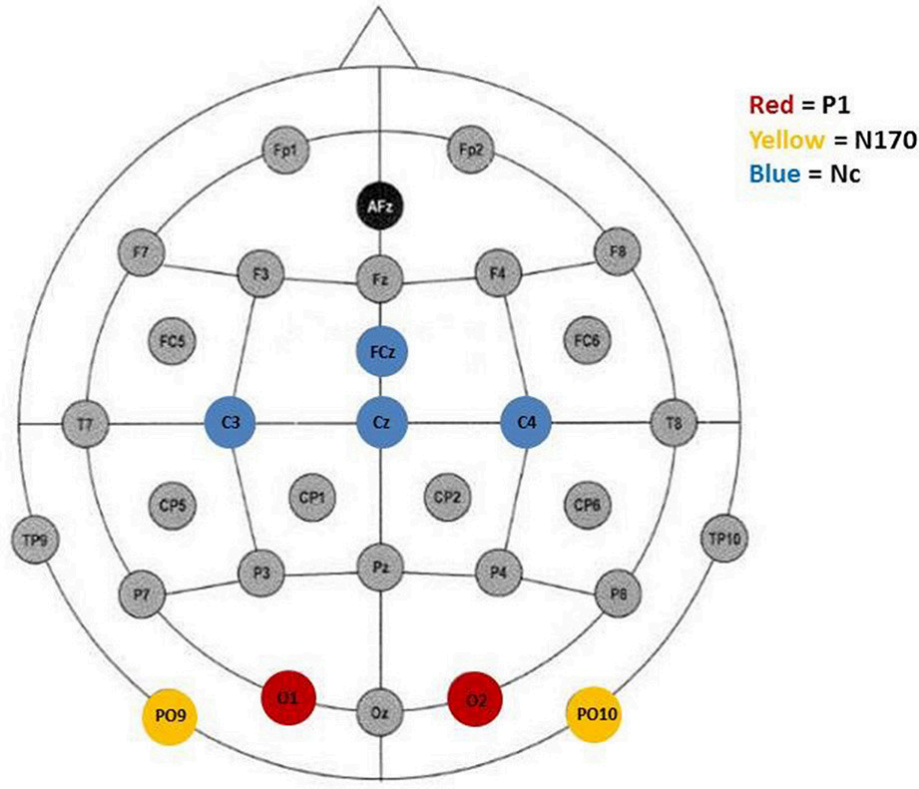
Electrode placement according to the 10–20 system. Colored electrode sites represent leads included in the analyses of P1 (red), N170 (yellow), and, Nc (blue).

Peak detection was performed automatically. It was informed by inspection of the grand average waveforms and previous literature. The P1, indicated by a positive deflection, was defined as the maximum positive peak at occipital sites (O1/2) occuring between 80 and 220 ms (*M* = 167, *SD* = 27). Furthermore, the N170 was defined as the most negative peak distributed over posterior electrode sites (PO9/10) occurring between 210 and 370 ms after stimulus onset (*M* = 307, *SD* = 31). Finally, in accordance with the literature, the Nc was defined as the second most negative peak at fronto-central electrode-sites after stimulus onset occuring between 280 and 520 ms (*M* = 390, *SD* = 27). Previous studies with young children have found the Nc to be prominent over distributed scalp regions and have included a number of electrodes (e.g., Carver et al., 2003; Swingler et al., 2007). In the current study, the Nc was analyzed at the midline (FCz, Cz) as well as the lateral lead pair C3/4. Figure 1 shows electrode sites representing leads that were included in the analyses.

### Analysis plan and statistical considerations

Differences in foster children and control children as well as effects of attachment security on (foster) children's facial familiarity processing, were analyzed for P1, N170, and Nc separately. Therefore, brain electrical activity in response to mother and stranger faces were separately subjected to a repeated measure design. The ANOVAs were conducted with foster care status (foster group, control group) and attachment (secure, insecure) as the between-subjects factors, and face type (mother, stranger) as the within-subjects factor. Also, hemisphere (for lateral lead pairs) or lead (for midline leads) was added as an additional repeated factor. To investigate whether foster children's (or control children's) brain activity in response to mother and stranger faces may vary as a function of attachment, three-way interaction effects between attachment, foster care status and face type, were considered within the main analyses. As ERP amplitude responses were not correlated with age, and control and foster children did not differ regarding age at the time of the ERP assessment, it was not necessary to include age as a covariate in the analyses. To reveal interaction effects, LSD pairwise comparisons were performed. Note that mean values reported within descriptions of the main analyses (including **Table 2**) are estimated marginal means and standard errors.

## Results

### Attachment security

Across the total sample attachment security ranged from −0.10 to 0.60 (*M* = 0.32, *SD* = 0.19) with a median of *Md* = 0.35. *T*-Tests showed that there was no difference between foster children and control children regarding attachment security, *t*_(35)_ = 0.22, ns. To compare children scoring high on attachment security with those who were rated less securely attached the total sample was split at the median leading to two groups. Table 1 shows that the distribution of children assigned to insecure or secure^3^ was comparable in foster and control children.

**Table 1:** AQS security score and frequency of securely and insecurely attached children by group in numbers. Pearson's Chi^2^ test results.

**Attachment group**	**AQS security score**	**Foster group**	**Control group**	**Total sample**	**Chi^2^**	***p***
Insecure	≤ 0.35	8	11	19	0.83	ns
Secure	>0.35	5	13	18		
Total sample		13	24	37		

### ERP responses to mother and stranger faces with regard to foster care status and attachment

#### P1 amplitude responses

For P1 amplitude responses the 2 face type × 2 foster care status × 2 attachment × 2 hemisphere repeated measures ANOVA with P1 amplitude responses as the dependent variable revealed no significant effects, which means that the P1 was unaffected by facial familiarity, neither did foster and control children nor securely attached from insecurely attached children differ regarding P1 amplitude responses to both faces.

#### N170 amplitude responses

**Figure 2:**
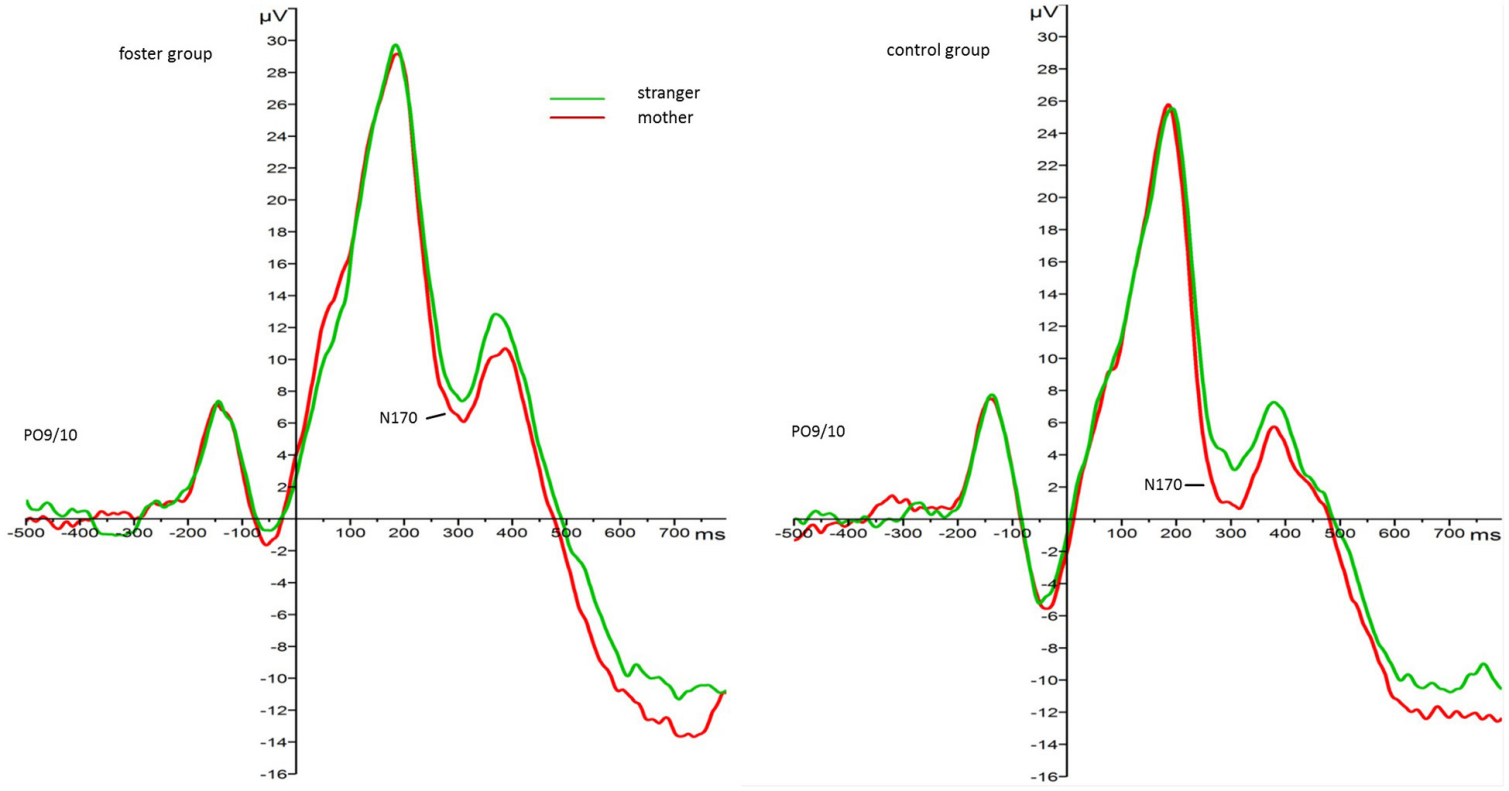
Grand average waveforms for N170 amplitude response (microvolt) to mother (red) and stranger faces (green) by foster care status. Collapsed over parieto-occipital leads PO9 and PO10.

The 2 face type × 2 foster care status × 2 attachment × 2 hemisphere repeated measures ANOVA with N170 amplitude as the dependent variable revealed main effects for face type, *F*_(1, 33)_ = 4.88, *p* = 0.034, $\eta_{p}^{2}=$ 0.13, foster care status, *F*_(1, 33)_ = 7.00, *p* = 0.012, $\eta_{p}^{2}=$ 0.18, attachment, *F*_(1, 33)_ = 6.33, *p* = 0.017, $\eta_{p}^{2}=$ 0.16, but no interaction effects. Regarding the main effect for face type, mother faces elicited larger amplitudes than stranger faces in all children (*M*_*mother*_ = −0.02, *SE* = 1.03.53; *M*_*stranger*_ = 2.09, *SE* = 1.13). Regarding the main effect for foster care status mean N170 amplitude responses were larger (more negative) in the control group (*M* = −1.87, *SE* = 1.16) than in the foster group (*M* = 3.94, *SE* = 1.57). Figure 2 depicts the grand average waveforms for each group in response to mother and stranger faces^4^.

**Figure 3:**
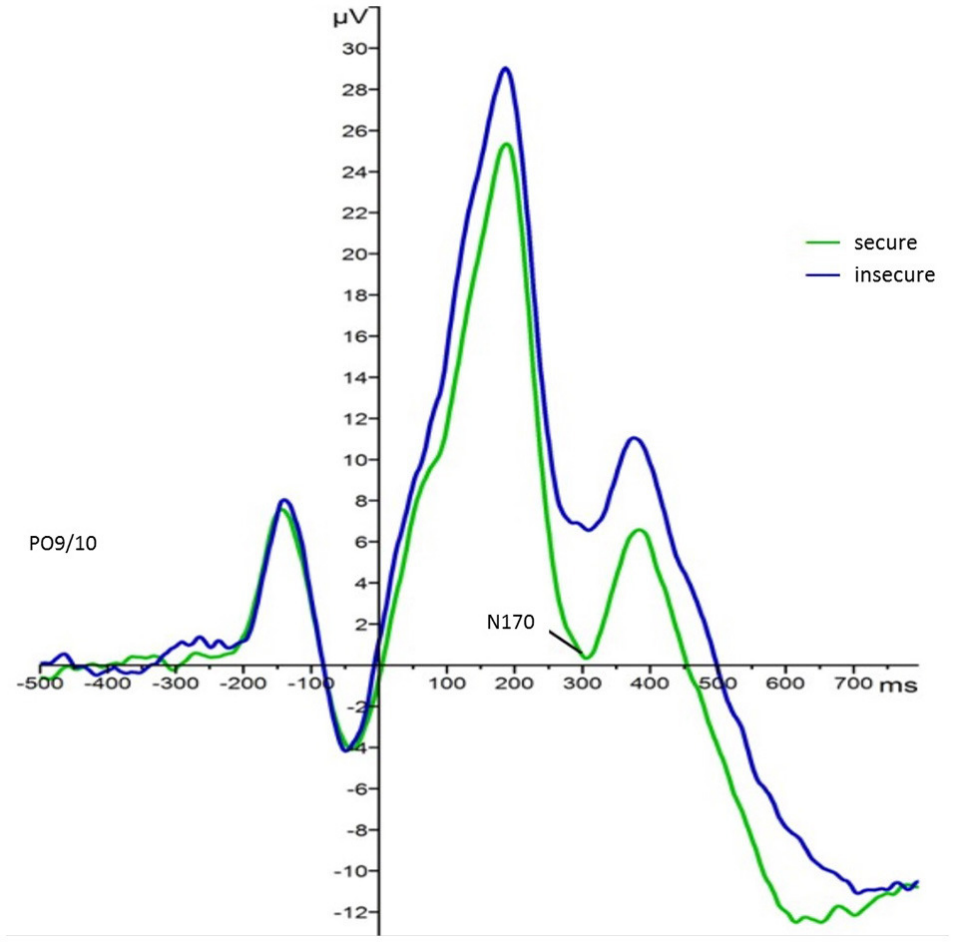
Grand average waveforms for N170 amplitude responses (microvolts) in securely (green) and insecurely (blue) attached children collapsed over parieto-occipital electrodes PO9 and PO10, face type, and foster care status.

For the main effect of attachment, mean values indicated that regardless of face type and foster care status securely attached children elicited larger N170 responses (*M* = −1.60, *SE* = 1.41) than insecurely attached children (*M* = 3.12, *SE* = 1.24). This effect is visualized in Figure 3,^5^. Also, N170 amplitude responses to both faces were larger on the left (*M* = −0.28, *SE* = 1.05) than on the right hemisphere (*M* = 2.35, *SE* = 1.20).

#### Nc amplitude responses

To investigate our research questions with regard to Nc amplitude responses, two separate analyses were conducted, first, for central lateral leads (C3/C4), and second, for fronto-central midline leads (FCz/Fz). In line with previous literature (e.g., Swingler et al., 2007) lateral leads were analyzed separately to include potential hemisphere effects.

**Figure 4:**
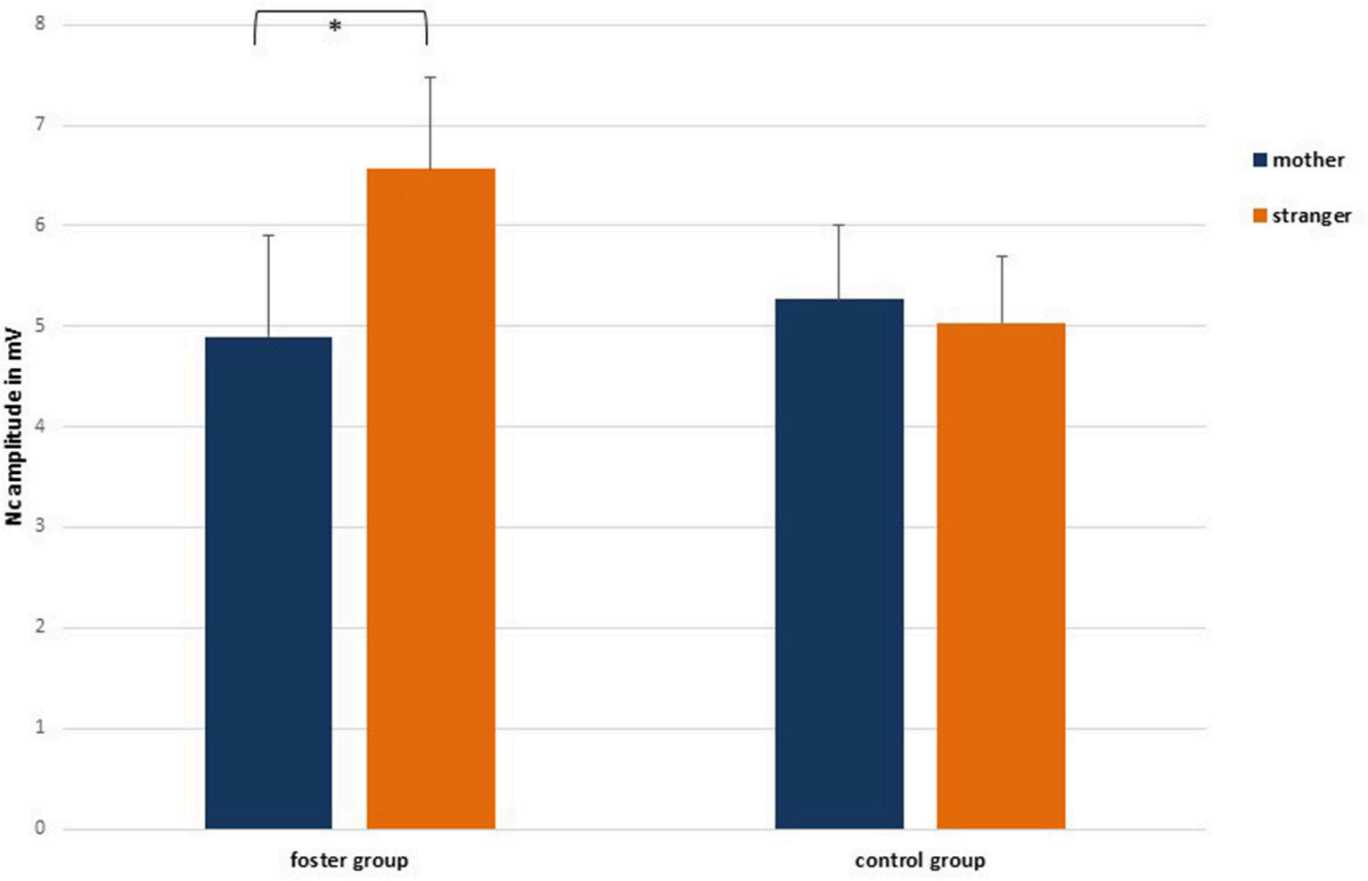
Nc amplitude responses in the mother and stranger face condition by group, collapsed over central lateral leads C3/4. Means and standard errors. Note that scale is inverted. ^*^p < 0.05

At the central lateral lead pair C3/4 the 2 face type × 2 foster care status × 2 attachment × 2 hemisphere repeated measures ANOVA showed a significant main effect for hemisphere, *F*_(1, 33)_ = 9.33, *p* = 0.004, $\eta_{p}^{2}=$ 0.22, and a significant interaction between foster care status and face type, *F*_(1, 33)_ = 6.39, *p* = 0.02, $\eta_{p}^{2}=$ 0.16. Regarding the main effect for hemisphere, mean values were larger on the left (*M* = −6.20, *SE* = 0.57) than on the right hemisphere (*M* = −4.66, *SE* = 0.66). For the interaction between foster care status and face type, Figure 4 indicated and pairwise comparisons confirmed that at central lateral leads the foster group showed larger Nc responses in the stranger face condition (*M* = −6.56, *SE* = 0.92) than in the mother face condition (*M* = −4.86, *SE* = 1.01), *p* = 0.016, whereas the control group did not significantly discriminate between the two faces at central lateral leads (*M*_*mother* =_ −5.27, *SE* = 0.74; *M*_*stranger*_ = −5.03, *SE* = 0.67).

At midline leads (Cz/FCz), the 2 face type × 2 group × 2 attachment × 3 lead repeated measures ANOVA solely revealed a highly significant main effect for lead, *F*_(1, 33)_ = 12.12, *p* = 0.001, $\eta_{p}^{2}=$ 0.27, with Nc amplitude responses being more negative at FCz (*M* = −6.84, *SE* = 0.65) than Cz (*M* = −5.33, *SE* = 0.68).

**Figure 5:**
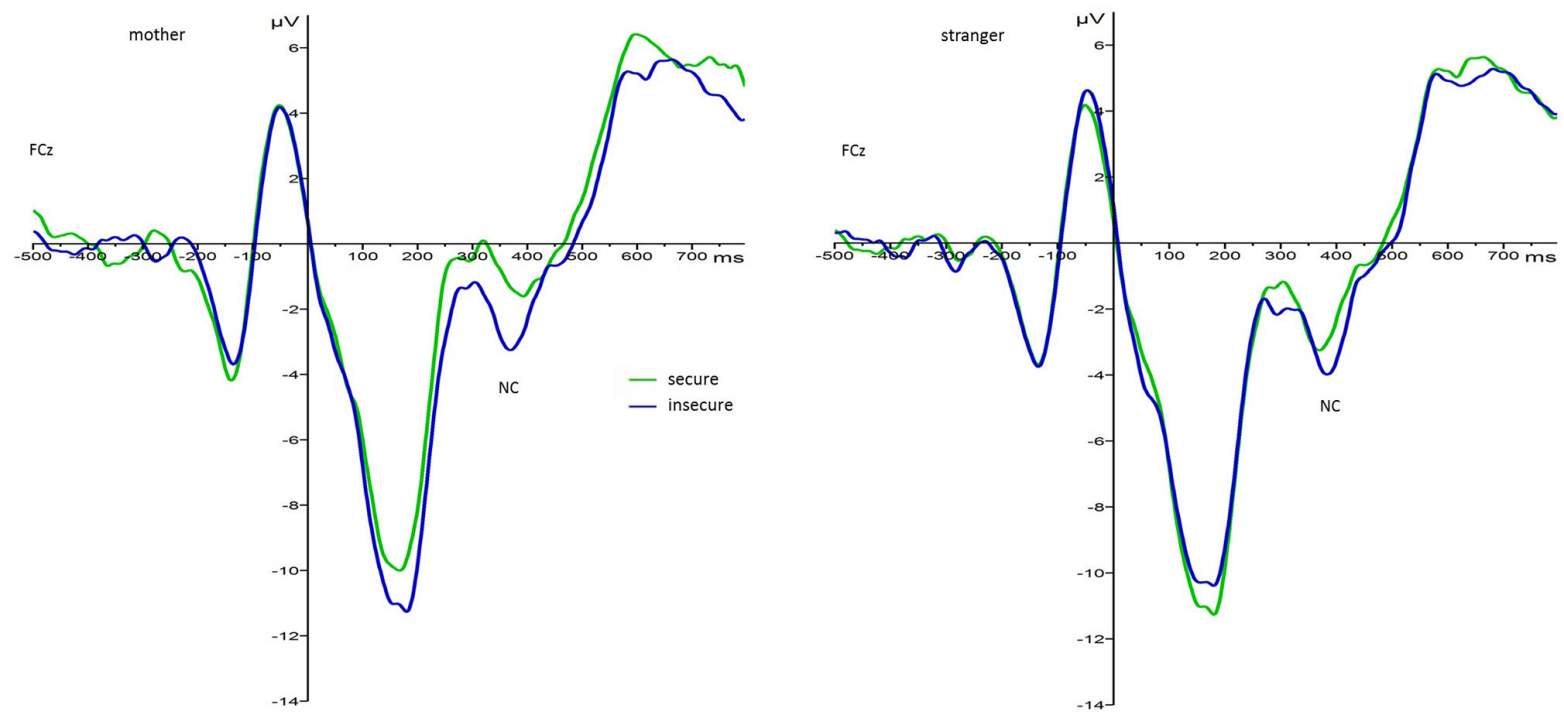
Grand average waveforms for Nc amplitude responses (microvolts) in securely (green) and insecurely (blue) attached children collapsed over electrodes Cz/FCz and groups by face type.

There also was a significant interaction effect between foster care status and face type, *F*_(1, 33)_ = 5.37, *p* = 0.03, $\eta_{p}^{2}=$ 0.14, as well as attachment and face type, *F*_(1, 33)_ = 5.27, *p* = 0.03, $\eta_{p}^{2}=$ 0.14. Regarding the interaction between foster care status and face type, pairwise comparisons showed that the difference between Nc amplitudes to mother as compared to stranger faces was only significant in the foster group with stranger faces (*M* = −7.63, *SE* = 1.18) eliciting larger amplitudes than mother faces (*M* = −5.22, *SE* = 1.04), *p* = 0.01. There was no difference in Nc amplitude responses to mother and stranger faces in the control group (*M*_*mother*_ = −5.70, *SE* = 0.74; *M*_*stranger*_ = −5.55, *SE* = 0.84). This resembles the interaction effect found at the central lead pair. For the significant interaction between attachment and face type, pairwise comparisons showed that Nc amplitude responses to mother faces varied as a function of attachment security (see Figure 5). More precisely, securely attached children elicited smaller Nc amplitudes to their mother's face (*M* = −3.80, *SE* = 0.96) than insecurely attached children (*M* = −7.12, *SE* = 0.84), *p* = 0.014. Regarding Nc amplitude responses to stranger faces the difference between insecurely and securely attached children did not reach significance (*M*_*insecure*_ = −6.98, *SE*_*insecure*_ = 0.96; *M*_*secure*_ = −6.19, *SE*_*secure*_ = 1.09). Also, pairwise comparisons between mother and stranger faces by attachment status showed that only in securely but not insecurely attached children the difference in Nc amplitude responses to mothers vs. stranger faces was significant, *p* = 0.007.

### Three-way interactions between foster care status, attachment and face type

In an exploratory manner we investigated whether differences in amplitude responses to faces varied as a function of the interaction between foster care status and attachment being aware that this analysis is based on very small cell sizes.

The three-way interaction between attachment, foster care status and face type, did not reach significance neither for P1 nor for N170 amplitude responses to faces. Regarding the Nc, the effect was not significant at the central lateral lead pair. However, at fronto-central midline leads, we found the interaction between attachment, face type and foster care status, *F*_(1, 33)_ = 6.43, *p* = 0.02, $\eta_{p}^{2}$ = 0.16, to be significant, and thus, qualifying the above described interaction effects between attachment and face type, as well as foster care status and face type at the midline leads. More precisely, pairwise comparisons suggested that the effect of attachment security on mother face processing was especially prominent in the foster group, *p* = 0.037, while it did not reach significance in the control group. As Table 2 shows, for the foster group Nc amplitudes to mother faces were more negative in insecurely than in securely attached children. Still, results should only be treated cautiously.

**Table 2:** Nc amplitude responses at midline leads in the control group and the foster group by face type and attachment. Means and standard errors.

**Face**	**Attachment security**	**Group**	
		**Control group** **Mean *(SE)***	**Foster group** **Mean *(SE)***
Mother	Insecure	−6.78 (1.10)	**−7.47 (1.29)**
	Secure	−4.63 (1.01)	**−2.98 (1.63)**
Stranger	Insecure	−6.75 (1.24)	−7.21 (1.46)
	Secure	−4.34 (1.14)	−8.05 (1.85)

*Values that significantly differ on a p = 0.05 level when compared pairwise are shown in bold*.

## Discussion

In this study, we asked how early caregiving experiences affect neural correlates of facial familiarity processing, which has been shown to be an important biological marker of children's social-emotional development. The group of foster children included in this study showed the same level of attachment security 1 year after placement as the control group resembling previous studies with the same (Lang, 2014) as well as other samples of children placed in foster or adoptive care (see Van den Dries et al., 2009).

In accordance with empirical evidence on the time course of facial familiarity processing we found effects of facial familiarity to occur at occipito-temporal electrode sites as early as the N170 time window, as well as at fronto-central sites during the later Nc time window, but not the P1 component. As the P1 is known to be based on “low-level visual information” (Rossion and Jacques, 2012, p. 127) this finding confirms that (foster) mother and stranger faces did not substantially differ in terms of basic visual cues like, for instance, luminance or contrast, which further validates the use of the stimuli.

### Neural responses to faces with regard to foster care status, attachment security and facial familiarity

#### P1 amplitude responses

In the current study, foster children and control children did not differ regarding P1 amplitude responses. Previous studies with children with a history of institutionalization, however, have found dampened P1 amplitude responses to faces, which has been interpreted as a sign of cortical hypo-arousal due to experiences of early deprivation (Moulson et al., 2009; Mesquita et al., 2015). There may be several explanations for these diverse findings. First, foster children's previous experiences may not be comparable to those of institutionalized children, most of whom have been affected by social deprivation from early on. This does not mean that neglect within a family environment is considered less severe; however, the heterogeneity of previous experiences in our foster sample may have obscured the effect of early deprivation on the P1 amplitude response. Second, findings from one study suggest that even within a group of institutionalized children only a subgroup showed dampened P1 amplitudes which was associated with atypical social behavior (Mesquita et al., 2015). Thus, different mechanisms underlying children's heterogeneous behavioral organization may have obscured effects in our sample. Third, the time living within the foster home may have been sufficient for a catch-up. This explanation is supported by findings from Moulson et al. (2009) who found the P1 (and other component's) amplitude responses in an intervention group to fall in between institutionalized and control children. Future research including larger sample sizes are needed to further reveal conditions under which subgroups are affected by what has been interpreted as cortical hypo-arousal.

In the same line, we did not find attachment security to affect face processing as early as the P1 time window. In concordance with the above non-findings of effects of foster care status at this early stage, it is suggested that children in our sample may have had experienced a “good enough” environment to develop such basic visual processing abilities (see Winnicott, 1973).

#### N170 amplitude responses

As expected we found both groups to differentially process faces as early as the N170 time window. Interestingly, in our sample, N170 amplitude responses were clearly dampened for foster children as compared to control children, suggesting an effect of possible hypo-arousal, yet, at a later processing stage. The N170 amplitude was affected by facial familiarity and attachment security with amplitudes being smaller, or less negative, in the stranger face condition than in the mother face condition, and, in insecurely attached as compared to securely attached children.

##### Differences between foster and control children

We found distinct group differences in facial processing regardless of face type. In our study, foster children as compared to control children elicited smaller amplitudes to both types of faces. Indeed, foster children's N170 amplitude responses did not fall below the zero level. Previous studies have shown smaller amplitudes in a series of face-sensitive components when comparing early socially deprived children with control children (Moulson et al., 2009) as well as when comparing currently institutionalized children that show atypical vs. typical social behavior (Mesquita et al., 2015). In our study, however, only the N170, but not the P1, was affected. Thus, the extent to which early facial processing stages are impaired may depend on specific sample characteristics. Consequently, in most foster children the early home environment or recent experiences within the foster home may have provided sufficient sensory input to acquire basic perceptual skills for a normal P1 function. In contrast, the N170, based on more elaborate processing, may more depend on early contingent face to face interactions, which may be restricted in foster children's early experiences. Thus, foster children's experiences may have led to a less matured neural network that in turn respond to social cues with relative hypoactivation within the N170, but not the P1, time window.

Interestingly, studies with the Bucharest sample have shown that in institutionalized children basic perceptional skills like facial emotion and facial familiarity processing have evolved despite severe early deprivation (Moulson et al., 2009). Thus, it is possible, that brain activation is generally affected by early adverse experiences in terms of cortical hypoarousal as the neural system did not receive the expected overall social input, while the input, on the other hand, may still be enough to develop basic perceptual skills.

Importantly, cortical hypoactivation has shown to be related to atypical social behavior like social disinhibition in studies on global measures of brain activity (Tarullo et al., 2011) as well as in studies suggesting hypoactivation in face-sensitive brain regions (Mesquita et al., 2015). Integrating findings from the behavioral part of our analyses that show enhanced social disinhibition in foster children (Kungl, 2016) provides supporting evidence for this relationship.

##### Effects of facial familiarity

The familiarity effect is in line with our expectations as well as with other studies who found the N170 to be sensitive to facial familiarity and also to the salience of faces in children and adults (e.g., Caharel et al., 2002, 2006; Dawson et al., 2002; Todd et al., 2008; Wild-Wall et al., 2008; Moulson et al., 2009; Mesquita et al., 2015). Research suggests that facial familiarity affects early visually evoked potentials as it implies the activation of mental representations, which, by nature are stronger in familiar, and thus well-known, than in unfamiliar faces (Herzmann et al., 2004; Leibenluft et al., 2004; Wild-Wall et al., 2008; Taylor et al., 2009).

In our study familiar faces elicited larger N170 amplitude responses than unfamiliar faces in both groups, which is in line with previous findings (Caharel et al., 2006, 2011; Wild-Wall et al., 2008; Dai et al., 2014, but see Todd et al., 2008). Additional testing N170 latencies in our sample revealed the N170 amplitude response to be significantly faster in the mother face than in the stranger face condition (Kungl, 2016), which suggests a facilitated visual coding of (foster) mother faces possible due to the “extensive visual experience” children have with this stimulus (Rossion and Jacques, 2012, p. 113).

In contrast to our expectations but consistent with previous findings (e.g., Moulson et al., 2009), there was no modulation of the familiarity effect by foster care status, and thus, individual experiences (e.g., the amount time children have spent with their (foster) mother).

This finding may be explained referring to priming or repetition studies suggesting the facial familiarity effect to require relatively short familiarization with the stimulus (Jemel et al., 2003; Caharel et al., 2011; but see Schweinberger et al., 2002). Thus, it is indicated that recent long-term exposure (during the time in the foster home) rather than lifelong experiences with the foster mother's face was sufficient to elicit a familiarity effect. Future research on mother-stranger face processing should include personally irrelevant but familiarized faces to detangle effects of personal salience and priming on the N170 amplitude.

##### Effects of attachment security

As expected, the N170 amplitude response clearly differed between securely attached and insecurely attached children. More precisely, securely attached children showed higher N170 amplitude responses than insecurely attached children regardless of foster care status. However, as foster children have shown to elicit smaller N170 amplitude than control children the effect added up in insecurely attached foster children showing the smallest deflections during this elaborated stage of facial processing. In conclusion, we again found children's socio-emotional experiences to have a strong effect on face processing in general, however, not facial familiarity processing in particular.

Interpreting this finding in line with the above, it is suggested that contingent social interactions within an adequately stimulating social environment may result in the formation of robust mental representations associated with an elaborated neural network that forms in an adaptive experience-dependent process (e.g., Nelson, 2001; Herzmann et al., 2004). When being exposed to social stimuli (here: faces) an elaborated neural network gets activated which may reflect in increased N170 amplitudes. If this assumption is true, it could be said that securely attached children show an expertise in the processing of social stimuli. Future research should address this question by, for example, additionally conducting a facial recognition task. Furthermore, it would be interesting whether the effect is face-specific. To address this question, future research should include ERPs to faces as well as objects.

The current data does not allow to make any assumptions on the causality of the effect. Indeed, it is possible that children with less processing activity in response to social cues were less likely to develop secure attachments to their (foster) mother. In this sense, this group of children could also be viewed as less responsive to changes in the social environment in terms of susceptibility (Ellis et al., 2011), which in turn may have hindered socio-emotional development. However, this interpretation clearly needs further empirical support, and, to detangle cause and effect, longitudinal assessments would be necessary.

#### Nc amplitude responses

##### Effects of foster care status and familiarity

The Nc component has repeatedly been associated with enhanced attentional processing (e.g., Reynolds and Richards, 2005) and its response to stranger faces has been shown to be influenced by different aspects of the mother–child relationship (e.g., Carver et al., 2003; Swingler et al., 2010). As expected, the interaction between group and face was significant. More precisely, only foster children elicited larger amplitudes to the stranger face as compared to the mother's face. Regarding foster children's heightened attention toward strangers on the behavioral level this clearly makes sense. Indeed, not only did we find foster children including the same sample to be described as showing more disinhibited behavior (e.g., Zimmermann, 2015) but also to show increased proximity seeking to the foster mother and elevated levels of looking behavior and verbal initiations directed to an approaching stranger during a behavioral assessment (described in Kungl, 2016). Thus, our neurophysiological findings on increased Nc amplitude responses (implying enhanced attention) to stranger faces in foster children may be a neural correlate of high vigilance around strangers. To further validate this assumption we ran additional analyses including children's looking behavior directed to an approaching stranger (see above) and found that the more children were looking at the stranger throughout the interaction the faster they responded to a stranger face in the ERP experiment in terms of shorter Nc latencies (Kungl, 2016). We suggest that our increased Nc amplitudes in foster children may reflect a neural correlate of aberrant social behavior in children from adverse rearing backgrounds. However, this assumption needs to be verified and the causality of the effect should be addressed in further studies.

##### Effects of attachment security

At midline leads we found that in response to mother faces Nc amplitudes were significantly decreased in securely attached children irregardless of foster care status. As the Nc amplitude reflects attentional processing of salient stimuli (e.g., Todd et al., 2008), this finding suggests, that securely attached children allocate less attentional resources when “faced” with their mother, probably because her face is associated with an internalized secure base in these children (Bowlby, 1982). Also, as internal working models of attachment are indicative of the child's appraisal of their social partners (Bretherton et al., 1990) in insecurely attached children, the mother's face may represent a more ambiguous stimulus and increased Nc amplitudes may be reflective of an increased effort in the evaluation of the mother's face's meaning. The effect may then be indicative of the activation of a certain neural circuit underlying the insecure status in the sense of contradicting cognitive and emotional responses. In concordance with our finding, Carver et al. (2003) found Nc responses to the mother face to vary in conjunction with proposed changes in children's social and emotional development. They argued that it is only after the establishment of the attachment relationship with the mother as the primary caregiver, that children devote less attentional resources to her face. In this line, our results add to this argument by suggesting that individual differences in attachment security moderate the effect described by Carver et al. (2003) and further confirms that experiences with the attachment figure are associated with different regulatory strategies that reflect on a psychophysiological level (Spangler and Grossmann, 1993). Future research may even be able to further reveal differences in mother's face processing when differentiating between ambivalent and insecure-avoidant attached children.

In addition to this two-way interaction between attachment security and face type there was a triple interaction between attachment, face type and group at midline leads. It suggested that the effect between attachment and face type was especially prominent in foster children. Looking at the means it is also obvious that large Nc amplitude responses to stranger faces were especially robust regarding effects of attachment security in foster children. This makes sense, as behavioral studies in children from adverse rearing environments have shown, stranger sociability to be rather independent from attachment security (e.g., Chisholm, 1998; Smyke et al., 2002; Zimmermann, 2015). Importantly, due to small cell sizes, it is unclear, if the 3-way interaction really qualifies the interaction between attachment and face type described above. Thus, this effect should only be interpreted cautiously and clearly needs replication including a larger sample of foster children. This would optimally provide the possibility of analyzing differences in Nc amplitude responses to stranger faces with regard to marked signs of atypical social behavior. Indeed, an ERP study comparing institutionalized children with atypical behavior to those with typical behavior, suggests that not all children's neural processing is affected by early experiences of pathogenic care in the same way (Mesquita et al., 2015).

### Limitations

There are several limitations to the study. First, we included 37 children, but the number of foster children was relatively small (*n* = 13). This uneven distribution was due to a relatively high drop out in the foster care sample. In a drop-out analysis, however, we showed that the remaining sample of foster children did not differ from the original sample, neither in age, nor in any of the central study variables, nor in mental health status. Future studies need to include a larger sample of foster children.

Furthermore, it should be considered that in foster children, attachment security was assessed about 13 months after placement while the ERP assessment took place at an average of 21 months within the foster home. Still, we did not assume changes in foster children's attachment security during this time window for two reasons. First, longitudinal data with the same sample indicated that the attachment relationship between foster mother and child formed within the first months of placement. More precisely, attachment security increased during the first 6 months in the foster home and then remained relatively stable (Lang, 2014). And second, previous studies suggest a general stability of attachment security during childhood (Main et al., 1985; Wartner et al., 1994).

The next limitation concerns the age range of our sample. Notably, neural structures develop rapidly during the early years and 3–6 years may be too wide to capture important developmental aspects of facial familiarity processing. As it was shown by Carver et al. (2003), the neural processing of the mother's and a stranger's face underlies significant changes over the preschool years. In the current study, however, we focused on group differences in two age-matched samples and thus, we assume our results to be due to different relational experiences rather than age effects.

Furthermore, it has to be noted, that foster children are not a homogeneous sample in terms of their prior experiences, and, they have also different (sub-) clinical symptoms (Zimmermann, 2015). For example, it may be crucial to differentiate between disinhibited and inhibited as well as typical and atypical social behavior (see Mesquita et al., 2015). Again, heterogeneity in our sample may have obscured specific processing patterns that are crucial to our understanding of different pathways leading to distinct behavioral outcomes. In line with this argument, it could be very informative to include a categorical measure of attachment that distinguishes between insecure-avoidant and insecure-ambivalent attached children. Especially, with regard to attentional processing of familiar faces it could be assumed, that in insecure ambivalent—as compared to insecure avoidant—the use of hyperactivating strategies may be evident even on a neural level. Still, with regard to our study aims and sample characteristics (e.g., age range, sample size) using a dimensional measure was the better choice.

Also, it has to be noted, that our results are correlational and that we cannot make any statement about the direction of effects. In the particular case of foster children, effects of intervention are of major interest. It has been shown that improvements in the caregiving environment have positive effects on a behavioral (e.g., Gabler et al., 2014; Lang, 2014) as well as on a neurophysiological level (Moulson et al., 2009). Thus, future studies may include more than one measuring point assessing ERPs in relation to aspects of the attachment relationship, first, at time of placement, and second, after a certain amount of time spent within the foster home. Also, future studies may benefit from including mother variables like sensitivity, that are directly related to improvements in attachment in foster children (Gabler et al., 2014).

An important methodological limitation refers to the design of the ERP experiment. As noted earlier, there seemed to be a visually evoked potential, probably due to the presentation of the fixation cross at −300 ms (see Figure 2), which may have affected subsequent components. Considering this limitation the baseline correction was applied to activity prior to the fixation cross. Still, it is unclear if decreased N170 amplitudes are due to differences in the processing of face stimuli *per se*. However, as the face-sensitive P1 component, which precedes the N170, did not show between subject effects we did not expect N170 effects to be due to prior processing differences. Nevertheless, results should be interpreted cautiously.

A final methodological limitation refers to the fact that we have only included female faces. In the current study caregivers were all female, however, with regard to reticence toward strangers and the processing of stranger faces, including male caregiver faces and male stranger faces may provide a more precise picture of the child's inner organization with regard to its actual social environment.

### Summary

Applying a neurophysiological approach by comparing high and low risk samples, the current investigation could show that facial processing—that is fundamental to adequate psycho-social functioning—is particularly sensitive to early caregiving experiences. It was indicated that adverse rearing backgrounds affect the growing organism on multiple levels possibly compromising the child's flexible psycho-social adjustment in later stages of development. Integrating results regarding ERP responses to faces at different stages during the time course of facial familiarity processing, we found that recognizing a familiar face elicits an increased neural response as early as the N170 time window suggesting a strong mental representation. Furthermore, at this stage, foster children as well as insecurely attached children showed dampened amplitudes, suggesting that children in more benign caregiving environments have developed increased expertise in face processing possibly due to having experienced more frequent face to face interaction. Finally, it was not until the Nc time window, reflecting advanced cortical processing, that foster children and control children differed with regard to facial familiarity. Here, we found foster children to show enhanced attentional processing in response to stranger faces, which may be a correlate of their aberrant social behavior toward strangers. Our neurophysiological and behavioral findings (see Kungl, 2016) provide further evidence that individual behavioral responses occurring during mother-stranger interaction are related to facial familiarity processing in normative development as well as in children at risk. Such investigations are important as alterations in social information processing may have cascading effects on children's development (Cicchetti, 2002). Longitudinal studies are needed to test the assumption that changes in neural correlates of psycho-social functioning would go along with changes in social behavior. Here, effects of attachment based interventions within the foster home could be a major focus.

## Ethics statement

The original study was approved by the Ethics Commission of the German Psychological Association (GS07200, 9/12/2009). For the EEG assessment foster mothers (foster group) and mothers (control group) were informed about the study's aims and methods. In addition, they were informed that their participation is voluntary and that they can withdraw from it any time without stating reasons, and that the data were treated according to the data protection law, saved anonymously and was to be deleted at any point if requested. Also they were informed about the EEG assessment. No invasive methods were used. Foster mothers and mothers signed the informed consent form before participation.

## Author contributions

MK: concept, design, recruitment of participants, supervision, acquisition and analyses of neurophysiological data, statistical analyses, interpretation of results, drafting, critical review. IB: overall study design, recruitment of participants, supervision, reliability training for coders, acquisition and analyses of behavioral data. GS: overall study design, supervision and guidance during all stages of the research processes, statistical analyses and interpretation of results, critical review.

### Conflict of interest statement

The authors declare that the research was conducted in the absence of any commercial or financial relationships that could be construed as a potential conflict of interest.

## Footnotes

^1^In addition, further behavioral data was collected at the laboratory visit. For an overview see Kungl (2016).

^2^For reasons of simplicity we use the term “mother” in the following. However, for foster children, stimuli were foster mother's faces.

^3^Note that we use the terms secure and insecure for children scoring above and, respectively, below the sample median in the AQS attachment security rating. However, this may not necessarily resemble the traditional classification introduced by Mary Ainsworth (e.g., Ainsworth et al., 1978).

^4^Importantly, we are aware that there appears to be a visually evoked potential, probably due to the presentation of the fixation cross starting at −300 ms (see Figure 2). The graph indicates that this waveform, which emerged prior to stimulus presentation, differed between both groups. However, regarding the earlier positive peak preceding the N170 the difference between foster and control group was not significant. Thus, it is most likely that the effect is specific to face-sensitive processing represented by the N170 component.

^5^Note that the difference between attachment groups regarding the earlier positive peak preceding the N170 was not significant.

## References

[B1] Ainsworth M. D. S. Blehar M. C. Waters E. Wall S. (1978). Patterns of Attachment: A Psychological Study of the Strange Situation. Hillsdale, NJ: Erlbaum.

[B2] Bowlby J. (1982). Attachment and Loss: Attachment, 2nd Edn., Vol. I. New York, NY: Basic Books.

[B3] Bretherton I. Ridgeway D. Cassidy J. (1990). Assessing internal working models of the attachment relationship: an attachment story completion task for 3-year-olds, in Attachment in the Preschool Years: Theory, Research and Intervention, eds Greenberg M. Cicchetti D. Cummings M. (Chicago, IL: University of Chicago Press), 273–308.

[B4] Caharel S. Courtay N. Bernard C. Lalonde R. Reba,ï M. (2005). Familiarity and emotional expression influence an early stage of face processing: an electrophysiological study. Brain Cogn. 59, 96–100. 10.1016/j.bandc.2005.05.005 16019117

[B5] Caharel S. Fiori N. Bernard C. Lalonde R. Rebaï M. (2006). The effects of inversion and eye displacements of familiar and unknown faces on early and late-stage ERPs. Int. J. Psychophysiol. 62, 141–151. 10.1016/j.ijpsycho.2006.03.002 16678927

[B6] Caharel S. Jacques C. d'Arripe O. Ramon M. Rossion B. (2011). Early electrophysiological correlates of adaptation to personally familiar and unfamiliar faces across viewpoint changes. Brain Res. 1387, 85–98. 10.1016/j.brainres.2011.02.070 21362409

[B7] Caharel S. Poiroux S. Bernard C. Thibaut F. Lalonde R. Rebaï M. (2002). ERPs associated with familiarity and degree of familiarity during face recognition. Int. J. Neurosci. 112, 1499–1512. 10.1080/00207450290158368 12652901

[B8] Carver L. J. Dawson G. Panagiotides H. Meltzoff A. N. McPartland J. Gray J.. (2003). Age-related differences in neural correlates of face recognition during the toddler and preschool years. Dev. Psychobiol. 42, 148–159. 10.1002/dev.10078 12555279 PMC3640993

[B9] Chisholm K. (1998). A three year follow-up of attachment and indiscriminate friendliness in children adopted from romanian orphanages. Child Dev. 69, 1092–1106. 10.1111/j.1467-8624.1998.tb06162.x 9768488

[B10] Cicchetti D. (2002). The impact of social experience on neurobiological systems: illustration from a constructivist view of child maltreatment. Cogn. Dev. 17, 1407–1428. 10.1016/S0885-2014(02)00121-1

[B11] Cole S. A. (2005). Infants in foster care: relational and environmental factors affecting attachment. J. Reproduct. Infant Psychol. 23, 43–61. 10.1080/02646830512331330947

[B12] Courchesne E. Ganz L. Norcia A. M. (1981). Event-related brain potentials to human faces in Infants. Child Dev. 52:804. 10.2307/1129080 7285651

[B13] Dai J. Zhai H. Wu H. Yang S. Cacioppo J. T. Cacioppo S.. (2014). Maternal face processing in Mosuo preschool children. Biol. Psychol. 99, 69–76. 10.1016/j.biopsycho.2014.03.001 24631724

[B14] Dawson G. Carver L. Meltzoff A. N. Panagiotides H. McPartland J. Webb S. J. (2002). Neural correlates of face and object recognition in young children with autism spectrum disorder, developmental delay, and typical development. Child Dev. 73, 700–717. 10.1111/1467-8624.00433 12038546 PMC3651041

[B15] de Haan M. Belsky J. Reid V. Volein A. Johnson M. H. (2004). Maternal personality and infants' neural and visual responsivity to facial expressions of emotion. J. Child Psychol. Psychiatry 45, 1209–1218. 10.1111/j.1469-7610.2004.00320.x 15335341

[B16] de Haan M. Johnson M. H. Halit H. (2007). Development of face-sensitive event-related potentials during infancy, in Infant EEG and Event-Related Potentials, ed de Haan M. (Hove; New York: Psychology Press), 77–100.

[B17] de Haan M. Nelson C. A. (1997). Recognition of the mother's face by six-month-old infants: a neurobehavioral study. Child Dev. 68, 187–210. 10.1111/j.1467-8624.1997.tb01935.x 9179998

[B18] de Haan M. Nelson C. A. (1999). Brain activity differentiates face and object processing in 6-month-old infants. Dev. Psychol. 35, 1113–1121. 10.1037/0012-1649.35.4.1113 10442879

[B19] Dozier M. Stovall K. C. Albus K. E. Bates B. (2001). Attachment for infants in foster care: the role of caregiver state of mind. Child Dev. 72, 1467–1477. 10.1111/1467-8624.00360 11699682

[B20] Dykas M. J. Cassidy J. (2011). Attachment and the processing of social information across the life span: theory and evidence. Psychol. Bull. 137, 19–46. 10.1037/a0021367 21219056

[B21] Eimer M. (2000). The face-specific N170 component reflects late stages in the structural encoding of faces. Neuroreport 11, 2319–2324. 10.1097/00001756-200007140-00050 10923693

[B22] Ellis B. J. Boyce W. T. Belsky J. Bakermans-Kranenburg M. J. Van IJzendoorn M. H. (2011). Differential susceptibility to the environment: an evolutionary–neurodevelopmental theory. Dev. Psychopathol. 23, 7–28. 10.1017/S0954579410000611 21262036

[B23] Gabler S. Bovenschen I. Lang K. Zimmermann J. Nowacki K. Kliewer J.. (2014). Foster children's attachment security and behavior problems in the first six months of placement: associations with foster parents' stress and sensitivity. Attach. Hum. Dev. 16, 479–498. 10.1080/14616734.2014.911757 24785376

[B24] Greenough W. T. Black J. E. Wallace C. S. (1987). Experience and brain development. Child Dev. 58, 539–559. 10.2307/1130197 3038480

[B25] Halit H. de Haan M. Johnson M. H. (2000). Modulation of event-related potentials by prototypical and atypical faces. Neuroreport 11, 1871–1875. 10.1097/00001756-200006260-00014 10884035

[B26] Herzmann G. Schweinberger S. R. Sommer W. Jentzsch I. (2004). What's special about personally familiar faces? A multimodal approach. Psychophysiology 41, 688–701. 10.1111/j.1469-8986.2004.00196.x 15318875

[B27] Ijzendoorn M. H. Bakermans-Kranenburg M. J. (2004). Maternal sensitivity and infant temperament in the formation of attachment, in Theories of Infant Development, eds Bremner G. Slater A. (Malden, MA: Blackwell Publishing), 233–257.

[B28] Itier R. J. (2004). N170 or N1? Spatiotemporal differences between object and face processing using ERPs. Cereb. Cortex 14, 132–142. 10.1093/cercor/bhg111 14704210

[B29] Jacobsen H. Ivarsson T. Wentzel-Larsen T. Smith L. Moe V. (2014). Attachment security in young foster children: continuity from 2 to 3 years of age. Attach. Hum. Dev. 16, 42–57. 10.1080/14616734.2013.850102 24215159

[B30] Jemel B. Pisani M. Calabria M. Crommelinck M. Bruyer R. (2003). Is the N170 for faces cognitively penetrable? Evidence from repetition priming of mooney faces of familiar and unfamiliar persons. Cogn. Brain Res. 17, 431–446. 10.1016/S0926-6410(03)00145-9 12880913

[B31] Jonkman C. S. Oosterman M. Schuengel C. Bolle E. A. Boer F. Lindauer R. J. L. (2014). Disturbances in attachment: Inhibited and disinhibited symptoms in foster children. Child Adolesc. Psychiatry Ment. Health 8:21. 10.1186/1753-2000-8-21 25057289 PMC4107487

[B32] Kishiyama M. M. Boyce W. T. Jimenez A. M. Perry L. M. Knight R. T. (2009). Socioeconomic disparities affect prefrontal function in children. J. Cogn. Neurosci. 21, 1106–1115. 10.1162/jocn.2009.21101 18752394

[B33] Kuefner D. Heering A. De Jacques C. Palmero-Soler E. Rossion B. (2010). Early visually evoked electrophysiological responses over the human brain (P1, N170) show stable patterns of face-sensitivity from 4 years to adulthood. Front. Hum. Neurosci. 3:67. 10.3389/neuro.09.067.2009 20130759 PMC2805434

[B34] Kungl M. (2016). The Impact of Early Experiences on Behavioral and Neural Correlates of Psycho-Social Functioning: A Study on Attachment, Social Interaction and Facial Familiarity Processing in Foster Children and a Control Group. Dissertation, Friedrich-Alexander Universität Erlangen-Nürnberg.

[B35] Lang K. A. (2014). Foster Parents' Parenting Characteristics and Foster Children's Pre-Placement Experiences: Influence on Foster Children's Psychosocial Adjustment during the First Year in Foster Placement. Dissertation, Friedrich-Alexander Universität Erlangen-Nürnberg.

[B36] Lang K. Bovenschen I. Gabler S. Zimmermann J. Nowacki K. Kliewer J. (2016). Foster children's attachment security in the first year after placement: a longitudinal study of predictors. Early Child. Res. Q. 36, 269–280. 10.1016/j.ecresq.2015.12.019

[B37] Lawler J. M. Hostinar C. E. Mliner S. B. Gunnar M. R. (2014). Disinhibited social engagement in postinstitutionalized children: differentiating normal from atypical behavior. Dev. Psychopathol. 26, 451–464. 10.1017/S0954579414000054 24621789 PMC4406408

[B38] Leibenluft E. Gobbini M. I. Harrison T. Haxby J. V. (2004). Mothers' neural activation in response to pictures of their children and other children. Biol. Psychiatry 56, 225–232. 10.1016/j.biopsych.2004.05.017 15312809

[B39] Main M. Kaplan N. Cassidy J. (1985). Security in nfancy, childhood, and adulthood: a move to the level of representation. Monogr. Soc. Res. Child Dev. 50, 66–104. 10.2307/3333827

[B40] Marshall P. J. Fox N. A. Bucharest Early Intervention Project Core Group. (2004). A comparison of the electroencephalogram between institutionalized and community children in Romania. J. Cogn. Neurosci. 16, 1327–1338. 10.1162/0898929042304723 15532128

[B41] Mesquita A. R. Belsky J. Crego A. Fachada I. Oliveira P. Sampaio A. (2015). Neural correlates of face familiarity in institutionally reared children with distinctive, atypical social behavior. Child Dev. 86, 1262–1271. 10.1111/cdev.12371 25899924

[B42] Moulson M. C. Westerlund A. Fox N. A. Zeanah C. H. Nelson C. A. (2009). The effects of early experience on face recognition: an eventrelated potential study of institutionalized children in Romania. Child Dev. 80, 1039–1056. 10.1111/j.1467-8624.2009.01315.x 19630892

[B43] Nelson C. A. (1994). Neural correlates of recognition memory in the first postnatal year, in Human Behavior and the Developing Brain, eds Dawson G. Fischer K. W. (New York, NY: Guilford Press), 269–313.

[B44] Nelson C. A. (2001). The development and neural bases of face recognition. Infant Child Dev. 10, 3–18. 10.1002/icd.239

[B45] O'Connor T. G. Marvin R. S. Rutter M. Olrick J. T. Britner P. A. English Romanian Adoptees Study Team. (2003). Child-parent attachment following early institutional deprivation. Dev. Psychopathol. 15, 19–38. 10.1017/S0954579403000026 12848433

[B46] Oosterman M. Schuengel C. (2008). Attachment in foster children associated with caregivers' sensitivity and behavioral problems. Infant Ment. Health J. 29, 609–623. 10.1002/imhj.20198 28636248

[B47] Otero G. A. Pliego-Rivero F. B. Fernández T. Ricardo J. (2003). EEG development in children with sociocultural disadvantages: a follow-up study. Clin. Neurophysiol. 114, 1918–1925. 10.1016/S1388-2457(03)00173-1 14499754

[B48] Pears K. C. Bruce J. Fisher P. A. Kim H. K. (2010). Indiscriminate friendliness in maltreated foster children. Child Maltreat. 15, 64–75. 10.1177/1077559509337891 19502477 PMC2810349

[B49] Pechtel P. Pizzagalli D. A. (2011). Effects of early life stress on cognitive and affective function: an integrated review of human literature. Psychopharmacology 214, 55–70. 10.1007/s00213-010-2009-2 20865251 PMC3050094

[B50] Pine D. S. Mogg K. Bradley B. P. Montgomery L. Monk C. S. McClure E.. (2005). Attention bias to threat in maltreated children: implications for vulnerability to stress-related psychopathology. Am. J. Psychiatry 162, 291–296. 10.1176/appi.ajp.162.2.291 15677593

[B51] Ponciano L. (2010). Attachment in foster care: the role of maternal sensitivity, adoption, and foster mother experience. Child Adolesc. Soc. Work J. 27, 97–114. 10.1007/s10560-010-0192-y

[B52] Quinn P. C. Yahr J. Kuhn A. Slater A. M. Pascalils O. (2002). Representation of the gender of human faces by infants: a preference for female. Perception 31, 1109–1121. 10.1068/p3331 12375875

[B53] Reynolds G. D. Richards J. E. (2005). Familiarization, attention, and recognition memory in infancy: an event-related potential and cortical source localization study. Dev. Psychol. 41, 598–615. 10.1037/0012-1649.41.4.598 16060807 PMC1464099

[B54] Rossion B. Caharel S. (2011). ERP evidence for the speed of face categorization in the human brain: disentangling the contribution of low-level visual cues from face perception. Vision Res. 51, 1297–1311. 10.1016/j.visres.2011.04.003 21549144

[B55] Rossion B. Jacques C. (2012). The N170: Understanding the time course of face perception in the human brain, in The Oxford Handbook of Event-Related Potentials, eds Luck S. J. Kappenman E. S. (Oxford: Oxford University Press), 115–141.

[B56] Rutter M. Colvert E. Kreppner J. Beckett C. Castle J. Groothues C.. (2007). Early adolescent outcomes for institutionally- deprived and non-deprived adoptees. I : Disinhibited attachment. J. Child Psychol. Psychiatry 48, 17–30. 10.1111/j.1469-7610.2006.01688.x 17244267

[B57] Schölmerich A. Leyendecker B. (1999). Deutsche Übersetzung des Attachment behavior Q-Set, Revision 3.2. Bochum: University of Bochum.

[B58] Schweinberger S. R. Pickering E. C. Jentzsch I. Burton A. M. Kaufmann J. M. (2002). Event-related brain potential evidence for a response of inferior temporal cortex to familiar face repetitions. Cogn. Brain Res. 14, 398–409. 12421663 10.1016/s0926-6410(02)00142-8

[B59] Smyke A. T. Dumitrescu A. Zeanah C. H. (2002). Attachment disturbances in young children I: the continuum of caretaking casualty. J. Am. Acad. Child Adolesc. Psychiatry 41, 972–982. 10.1097/00004583-200208000-00016 12162633

[B60] Spangler G. Bovenschen I. Nowacki K. (2009). Bindungsentwicklung und psychosoziale Anpassung von Pflegekindern: Individuelle und soziale Einflussfaktoren. Antrag auf Gewährung einer Sachbeihilfe an die Deutsche Forschungsgemeinschaft (DFG).

[B61] Spangler G. Grossmann K. E. (1993). Biobehavioral organization in securely and insecurely attached infants. Child Dev. 64, 1439–1450. 10.2307/1131544 8222882

[B62] Spangler G. Zimmermann P. (1999). Attachment representation and emotion regulation in adolescents: a psychobiological perspective on internal working models. Attach. Hum. Dev. 1, 270–290. 10.1080/14616739900134151 11708227

[B63] Stovall-McClough K. C. Dozier M. (2004). Forming attachments in foster care: infant attachment behaviors during the first 2 months of placement. Dev. Psychopathol. 16, 253–271. 10.1017/S0954579404044505 15487595

[B64] Swingler M. M. (2008). Brain-Behavior Correlations during Proposed Transitions in the Mother-Child Relationship : An Examination of Behavior and Face-Processing in Six-Month-Olds and Toddlers. San Diego, CA: University of California.

[B65] Swingler M. M. Sweet M. A. Carver L. J. (2007). Relations between mother-child interactions and the neural correlates of face processing in 6-month-olds. Infancy 11, 63–86. 10.1207/s15327078in1101_3

[B66] Swingler M. M. Sweet M. A. Carver L. J. (2010). Brain-behavior correlations: relationships between mother-stranger face processing and infants' behavioral responses to a separation from mother. Dev. Psychol. 46, 669–680. 10.1037/a0018907 20438178 PMC3593116

[B67] Tarullo A. R. Garvin M. C. Gunnar M. R. (2011). Atypical EEG power correlates with indiscriminately friendly behavior in internationally adopted children. Dev. Psychol. 47, 417–431. 10.1037/a0021363 21171750 PMC3484164

[B68] Taylor M. J. Arsalidou M. Bayless S. J. Morris D. Evans J. W. Barbeau E. J. (2009). Neural correlates of personally familiar faces: Parents, partner and own faces. Hum. Brain Mapp. 30, 2008–2020. 10.1002/hbm.20646 18726910 PMC6870744

[B69] Taylor M. J. Batty M. Itier R. J. (2004). The faces of development: a review of early face processing over childhood. J. Cogn. Neurosci. 16, 1426–1442. 10.1162/0898929042304732 15509388

[B70] Taylor M. J. Edmonds G. E. McCarthy G. Allison T. (2001). Eyes first! Eye processing develops before face processing in children. Neuroreport 12, 1671–1676. 10.1097/00001756-200106130-00031 11409737

[B71] Taylor M. J. McCarthy G. Saliba E. Degiovanni E. (1999). ERP evidence of developmental changes in processing of faces. Clin. Neurophysiol. 110, 910–915. 10.1016/S1388-2457(99)00006-1 10400205

[B72] Taylor-Colls S. Fearon R. M. P. (2015). The effects of parental behavior on infants' neural processing of emotion expressions. Child Dev. 86, 877–888. 10.1111/cdev.12348 25676831

[B73] Todd R. M. Lewis M. D. Meusel L.-A. Zelazo P. D. (2008). The time course of social-emotional processing in early childhood: ERP responses to facial affect and personal familiarity in a go-nogo task. Neuropsychologia 46, 595–613. 10.1016/j.neuropsychologia.2007.10.011 18061633

[B74] Van den Dries L. Juffer F. van IJzendoorn M. H. Bakermans-Kranenburg M. J. (2009). Fostering security? A meta-analysis of attachment in adopted children. Child. Youth Serv. Rev. 31, 410–421. 10.1016/j.childyouth.2008.09.008

[B75] Van Den Dries L. Juffer F. Van IJzendoorn M. H. Bakermans-Kranenburg M. J. Alink L. R. A. (2012). Infants' responsiveness, attachment, and indiscriminate friendliness after international adoption from institutions or foster care in China: application of emotional availability scales to adoptive families. Dev. Psychopathol. 24, 49–64. 10.1017/S0954579411000654 22292993

[B76] Van Ijzendoorn M. H. Vereijken C. M. J. L. Bakermans-Kranenburg M. J. Marianne Riksen-Walraven J. (2004). Assessing attachment security with the attachment Q sort: meta-analytic evidence for the validity of the observer AQS. Child Dev. 75, 1188–1213. 10.1111/j.1467-8624.2004.00733.x 15260872

[B77] Wartner U. G. Grossmann K. Fremmer-Bombik E. Suess G. (1994). Attachment patterns at age six in south germany: predictability from infancy and implications for preschool behavior. Child Dev. 65, 1014–1027. 10.2307/1131301

[B78] Waters E. (1995). Appendix A: the attachment q-set (version 3.0). Monogr. Soc. Res. Child Dev. 60, 234–246. 10.2307/1166181

[B79] Waters E. Deane K. E. (1985). Defining and assessing individual differences in attachment relationships: Q-methodology and the organization of behavior in infancy and early childhood. Monogr. Soc. Res. Child Dev. 50:41 10.2307/3333826

[B80] Webb S. J. Jones E. J. H. Merkle K. Venema K. Greenson J. Murias M.. (2011). Developmental change in the ERP responses to familiar faces in toddlers with autism spectrum disorders versus typical development. Child Dev. 82, 1868–1886. 10.1111/j.1467-8624.2011.01656.x 22004249 PMC4141561

[B81] Wild-Wall N. Dimigen O. Sommer W. (2008). Interaction of facial expressions and familiarity: ERP evidence. Biol. Psychol. 77, 138–149. 10.1016/j.biopsycho.2007.10.001 17997008

[B82] Winnicott D. W. (1973). The Child, The Family, and The Outside World. London: Penguin Books.

[B83] Zeanah C. H. Scheeringa M. Boris N. W. Heller S. S. Smyke A. T. Trapani J. (2004). Reactive attachment disorder in maltreated toddlers. Child Abuse Negl. 28, 877–888. 10.1016/j.chiabu.2004.01.010 15350771

[B84] Zimmermann J. (2015). Symptoms of Disordered Attachment in High-Risk Populations: Prevalence, Risk-Factors, and Prevention. Dissertation, Friedrich-Alexander Universität Erlangen-Nürnberg.

